# Nodular malignant melanoma in vulvar skin without pigmentation: a case report

**DOI:** 10.1186/s12905-021-01422-1

**Published:** 2021-08-06

**Authors:** Jing Nie, Yan Li, Xue Shen, Yan Liu, Haipeng Shi, Yonghong Lu

**Affiliations:** 1 Department of Dermatology, Chengdu Second People’s Hospital, No.165 Caoshi Street, Qingyang District, Chengdu, 610000 Sichuan Province China; 2 Department of Pathology, Chengdu Second People’s Hospital, Chengdu, China

**Keywords:** China, Dermoscopy, Histopathology, Nodular melanoma, Polypoid melanoma

## Abstract

**Background:**

Polypoid nodule growing without apparent pigmentation on the vulvar skin usually reminds us of the diagnostic pitfall, which is commonly and mistakenly diagnosed as other types of tumors. Although there are several manifestations of amelanotic melanoma are known, these malignancies are usually pigmented because they are derived from melanocytes containing melanin. However, amelanotic melanomas are easily misdiagnosed or their diagnoses were commonly delayed due to lack of pigmentation. Therefore, a solitary polypoid nodule is worth noting and further reporting. Particularly, the clinical characteristics and outcomes of the solitary polypoid nodule are rare in Asian patients.

**Case presentation:**

We presented an interesting case of a 33-year-old female with a solitary polypoid nodule without apparent pigmentation on her vulvar skin. Her medical history was unclear, no ulcer was seen in the lesion area, and dermatoscopy was indicated a possible tumorous change, which has caught the attention of clinicians, and then further examined by the pathologist. The final diagnosis was nodular malignant melanoma (NM) (Breslow thickness 9.5mm, Clark level 4).

**Conclusions:**

Hence, though reviewing this case record, the relevant literature and NM-related materials, we suggest that the combination of skin imaging technology and histopathological examination could provide us a better understanding and reduce the possibility of misdiagnosis in clinic practice.

## Background

Skin melanoma has become the sixth most common cancer in the United States, but it is still a relatively rare disease in Asia. Early diagnosis is the best way to improve the prognosis in patients with melanoma. Unfortunately, early diagnosis of nodular malignant melanoma (NM) is particularly challenging given that NM patients often lack identifiable manifestations such as moles or freckles, it is difficult to make an accurate diagnosis at the early stage. Prognoses of patients with malignant melanoma diagnosed in China were still suboptimal. Compared with other melanoma subtypes, NM has been shown to have a higher growth rate, greater bioavailability and mitosis. [1–5] Hence, most of the patients have already been in the locally advanced stage at the time of diagnosis (i.e., stage II or above). Among many different subtypes of melanoma, two of the most common subtypes are acute mild melanoma and mucosal melanoma in the Chinese population.[6] Recent studies have shown that although NM accounts for 14–15 % of all cutaneous melanoma (CM) cases, it leads to more than 37–40 % of mortality in melanoma.[7, 8] Besides, increasing evidence indicates the unique place of NM in the clinic, considering its special manifestations and histopathology, thus the early diagnosis and prognosis of patients with NM is particularly important.[9].

## Case presentation

### Clinical findings

**Fig. 1:**
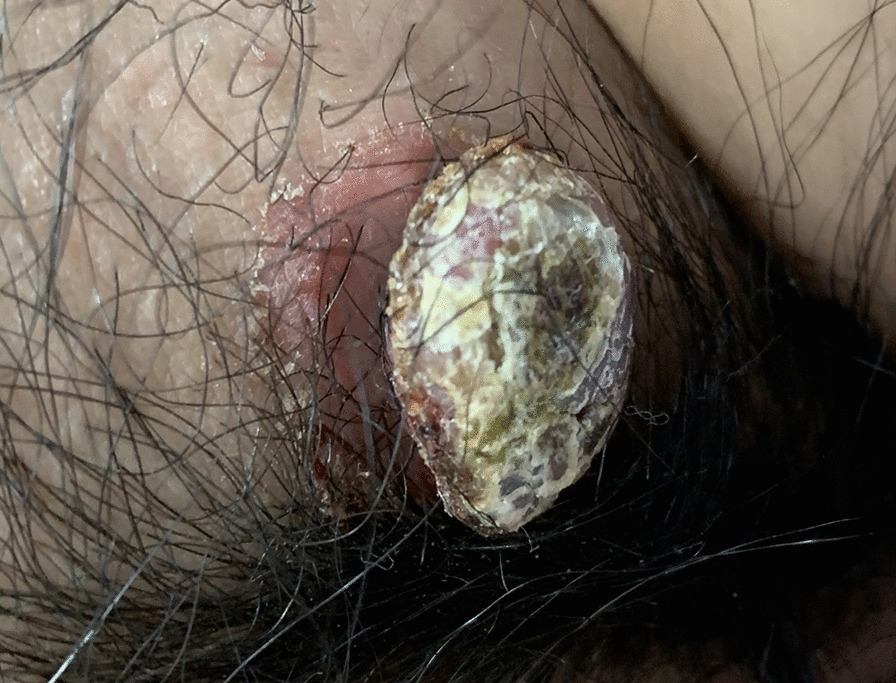
The red plaque was slightly bulgy, covered with a thick yellow-white crust. The plaque was 2.2 × 1.8 × 0.5 cm, and the polyp was 2.5 × 2 × 1.5 cm. The width of the pedicle was 1.4 cm

A 33-year-old female presented to the clinic complaining of a polyp on top of a red plaque in the left side mons pubis for 1 year. It was a kind of round flaky red bulge lesion in the skin of mons pubis. On top of this, it is shown sarcomatoid hyperplasia covering with the yellow-green crust, and the surrounding skin had no obvious abnormality and elcosis. The plaque was slightly bulgy without pressing pain, covered with a thick yellow-white crust (Fig. 1). The plaque was 2.2 × 1.8 × 0.5 cm, and the polyp was 2.5 × 2 × 1.5 cm. The width of the pedicle was 1.4 cm. There were no other systemic abnormalities or any palpable lymphadenopathy, ultrasound B was applied to evaluate the superficial inguinal lymph nodes. She had no significant history of medicine, surgery, irritation, and trauma. Before the lesion appearance, the patient had no discomfort, thus she did not pay attention to the skin lesion, and had not used other external medicines or ask for help from professionals. Until nearly a month, she found that the lesion was prone to bleed after friction, thus she came to our hospital for treatment. The lesion was removed surgically and the histopathologic examination was performed. The possibility of skin tumor was considered through dermoscopy, then the histopathological examination was performed to make the confirmation. Skin biopsy was made on December 31, 2019, and further immunohistochemical reports have been done on January 17, 2020. Detailed information was shown below.

### Dermatoscopy findings

**Fig. 2:**
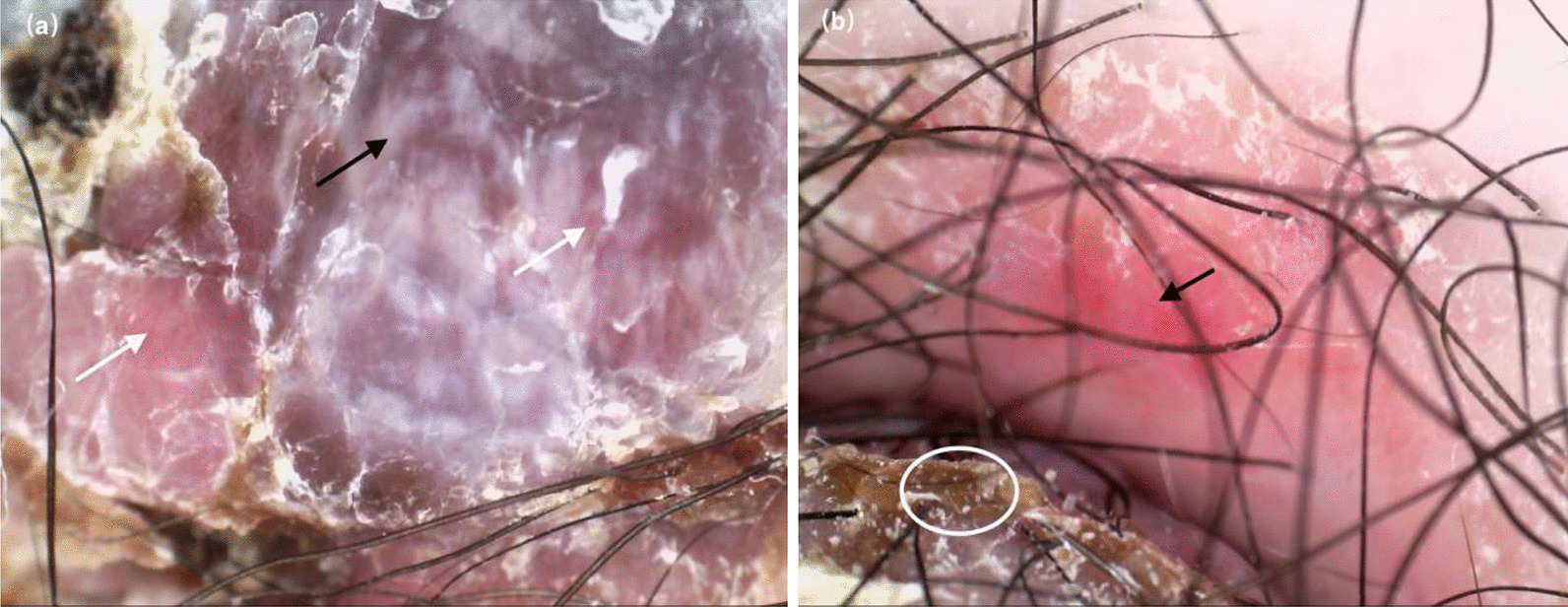
Dermoscopic findings in our patient: **a** There were spot-shaped and polymorphic vascular structures (white arrows), white homogeneous structures existing in the lesion (black arrows). **b** There were yellow-and-white crust (white circle), spot-shaped and polymorphic vascular structures (black arrows) presenting in the lesion (original magnifications:×30)

The dermoscopic images showed the dark red background, covering a thick yellow-and-white crust, with spot-shaped and polymorphic vascular structures which focally distributed. In some areas, white homogeneous structures could be seen, as well as dark red clumps. No typical pigment structure was seen (Fig. 2a, b). The skin lesions were considered as the diagnosis of skin tumors using dermoscopy.

### Histopathological findings

The excisional biopsy was carried out, and the specimen tissue was fixed in formalin, and embedded in paraffin. The histological examination showed: the skin lesion at the left side of mons pubis was spindle-shaped, the lesion size was 2.2 × 1.8 × 0.5 cm. There were gray-brown and mushroom-shaped protrusions on the epidermis, and the size of the protrusions was 2.5 × 2 × 1.5 cm, the pedicle width was 1.4 cm, and the transaction of the protrusions was grayish-white and grayish-red. Besides, Breslow thickness was about 9.5mm, and Clark level was IV. No tumor embolus was detected in the vessels. The tumor involvement had been found in the incisal edge of long-axis two sides and base of the tumor sample.

**Fig. 3:**
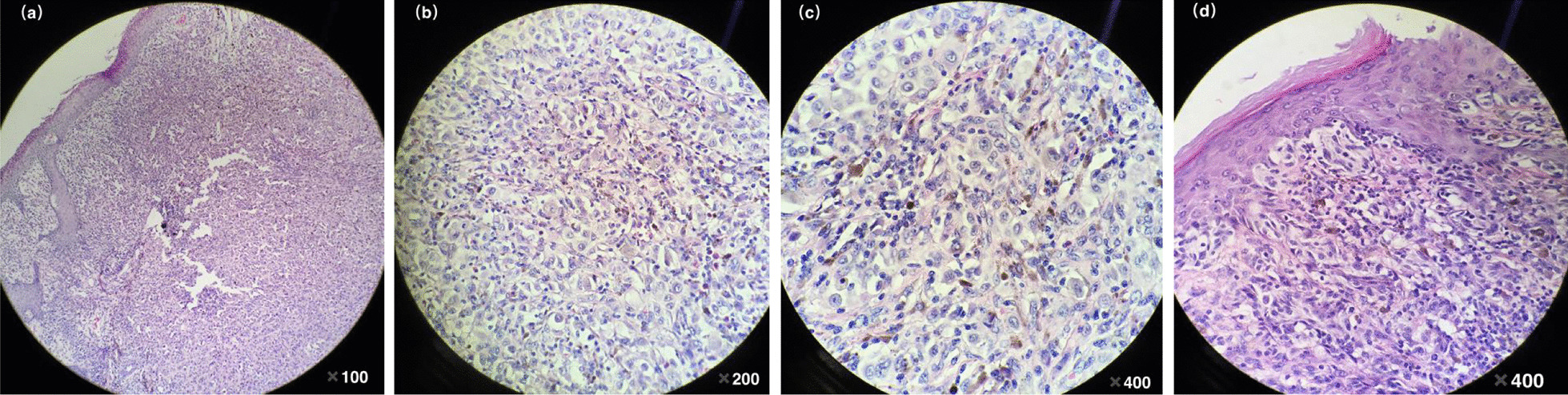
**a** Nodular growth of melanoma cells in the dermis. **b**, **c** Epithelioid tumor cells have obvious atypia, with vesicular nuclei, eosinophilic nucleoli, abundant cytoplasm, eosinophilia, and pigmentation in some cells. **d** The melanoma cells in the basal layer of the epidermis have Paget-like spread, and the dermal papilla is affected

Microscopically, the polyps were lined by melanocytes, with pale cytoplasm (Fig. 3a–d). It was represented as atypical cells and heteromorphic nuclei, with different cell sizes and abnormal mitosis of 5–7 counts /10HPF. Each slide was reviewed by 3 different pathologists, and the diagnosis was made as the melanocyte tumor.

The immunohistochemistry (IHC) studies revealed that the expressions of S-100, HMB-45, Melan A and Cyclin D1 were stained positive, while CD-117 was focally immunoreactive and CD31, P16, PCK and LCA were stained negative. The final histopathological diagnosis was made: melanocyte nevus malignancy, nodular malignant melanoma. This patient underwent a complete local excision and she recovered well recently.

### Follow-up

The patient was required to re-examined regularly after surgery, and recent follow-ups showed that she recovered well and there was no sign of recurrence till now.

## Discussion and conclusions

Melanoma is a highly aggressive cutaneous malignancy with considerable risk for metastasis. In dermatologic and diagnostic pathology, we divide melanomas into 4 subtypes based on the different characteristics of their histopathology, including diffuse surface, nodular, freckled, and terminal pigmented melanomas. These malignant tumors are typically pigmented given that they arise from melanocytes, who can produce melanin. Considering the disease’s metastasis and related mortality mainly depends on the thickness of the tumor and the depth of invasion, thus early detection and correct diagnosis are important to improve the prognosis of patients with melanoma. However, it is difficult to make an accurate diagnosis for melanoma at early stage in China [10].

In our present case, this patient had large polyps on her vulvar skin, but she did not look for help from professionals. Until nearly a month, she found that the lesion was prone to bleed after friction, so she came to our hospital for treatment.

According to the literature, it is reported that nodular melanoma generally has the following characteristics under dermatoscopy: peripheral black dots/globules, multiple brown dots, irregular black dots/globules, blue-white veil, homogeneous blue pigmentation, 5 to 6 colors, and black color, for instance, it was reported as milky red with homogeneous red structure; shiny white structure (crystalline structure); various pink colors; and irregular linear blood vessels [11].

It is well documented that NM often lacks pigmentation [12], which indicates that the diagnosis of NM cannot rely on the pigmentation in the tissue but vascular morphology [13]. The dermatoscopic findings indicated that the milky-red areas, short white shiny streaks (only seen under polarized dermoscopy) and polymorphic vascular morphology were the only clues for the diagnosis [12, 13]. The most common combination of blood vessel types in melanoma is linear, coiled, and spotted. Although short, shiny white streaks are not very specific for the diagnosis of NM, they are rarely seen in benign skin tumors, therefore, representing an important standard for NM treatment.

Back to our case, dermatoscopic images showed there was a dark-red background with short white shiny streaks, and the vessel types were linear, coiled, and spotted. Although no typical dermatoscopic changes were found, we tended to diagnose the lesion as a type of skin tumor.

Further performances such as histopathological analysis and IHC on the skin lesions of this patient were conducted. A variety of IHC staining biomarkers are widely accepted for the diagnosis of melanoma, including S-100, HMB-45 and Melan-A. S-100 is reported to be the most sensitive marker. Besides, specific HMB-45, S-100 and Melan-A are close to 100 %, 75–87 % and 95–100 %, respectively [14]. IHC analysis of this patient showed a strong positive of S-100 tumor cells protein, additionally, Melan A and HMB45 were all positive. Therefore, this case was confirmed as malignant melanoma nevus and nodular malignant melanoma formation.

Although NM accounts for 14–15 % of all CM patients, there are several different points between them, such as prognosis, mutational profile, and histopathologic characteristics. A previous clinic trial analyzed [15] 350 patients with vulvar melanoma from 6436 vulvar cases in the Dutch Cancer Registry between 1989 and 2012, the results of prognosis showed that the 5-year OS of women with vulvar melanoma was 35 % (95 % CI 26.7–44.4 %), compared to 50 % (95 % C=: 40.5–59.1 %) for matched-CM patients (p = 0.002). The prognostic factors of vulvar melanoma are mainly affected by Breslow’s thickness, AJCC tumor stage and lymph nodal status [16, 17]. In addition, the literature about mutational profiles of vulvar melanoma and CM demonstrated that BRAF mutations are most common (50 − 60 %) in CM, while c-KIT and NRAS mutations (27.6 %) commonly exist in vulvar melanoma [18]. The histologic features include atypical melanocytes, which mostly distributed in the basal layers. The invasive atypical melanocytes mainly distributed in nests, which could be epithelioid, spindle and other forms. Melanin pigment can be diffuse or focal and even absent. In China, polypoid nodular malignant melanoma on the vulvar skin is rare, and there is no accurate and practical diagnostic standard for this disease in the clinic. Hence, it is crucial to make the right diagnosis at the early stage of melanoma using dermoscopy or other approaches, performing histopathology and immunological examinations in time, and conducting the early detection and treatment to help these patients improve their prognosis.

## Data Availability

All patient data and clinical images adopted are contained in the medical files of the Dermatology Department of Chengdu Second People’s Hospital. The data supporting the conclusions of this article are included within the article and its figures and tables.

## Abbreviations

NM: Nodular malignant melanoma
ALM: Acute mild melanoma
CM: Cutaneous melanoma
